# Supplementary figures and images for: Real-time monitoring of peptidoglycan synthesis by membrane-reconstituted penicillin-binding proteins
Source: eLife. 2021 Feb 24;10:e61525. doi: 10.7554/eLife.61525 (PMC7943195; doi:10.7554/eLife.61525)

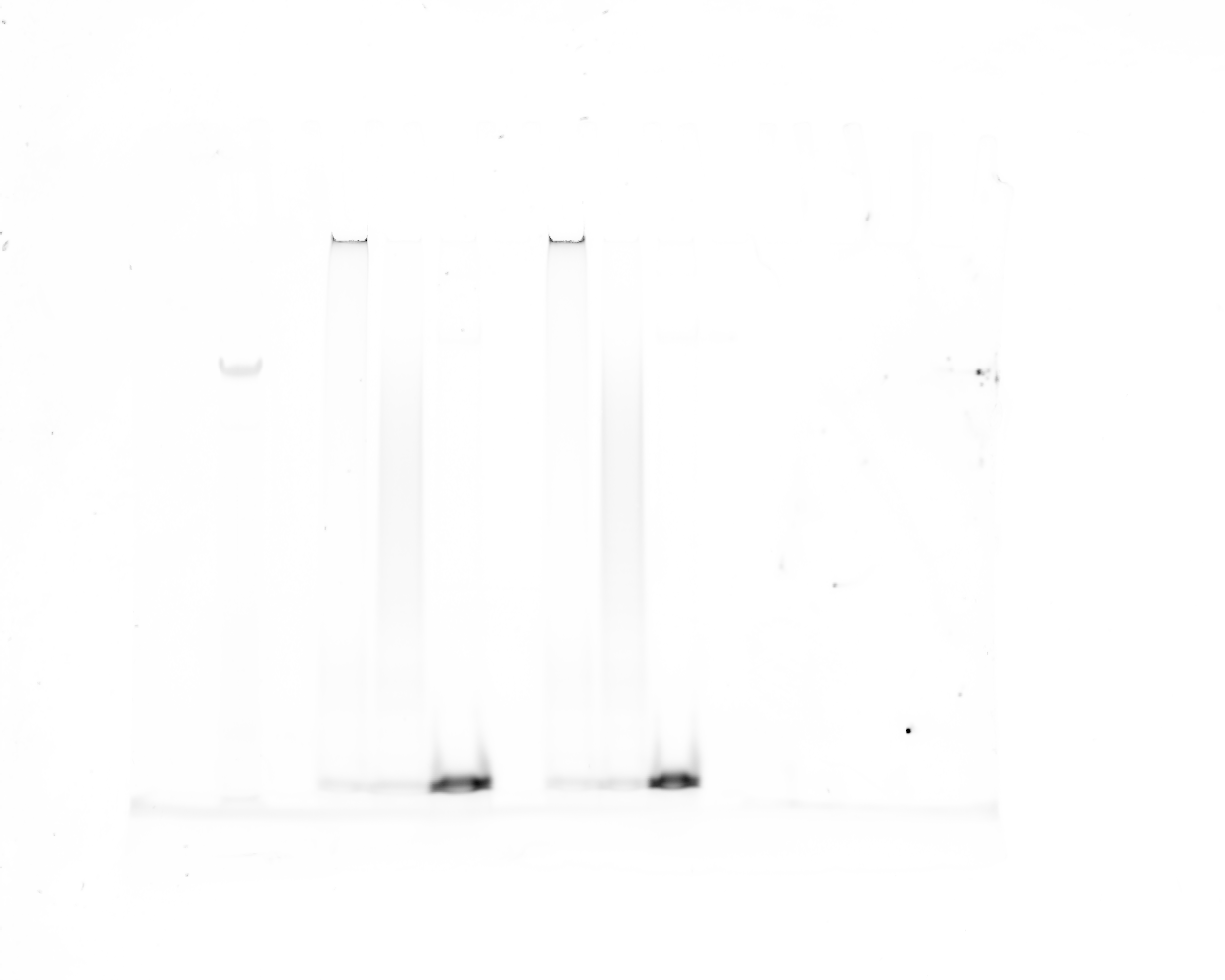

Supplement: Figure 1—source data 1. [file elife-61525-fig1-data1.zip › Figure 1 - panel B - Atto550 fluorescence.tif]

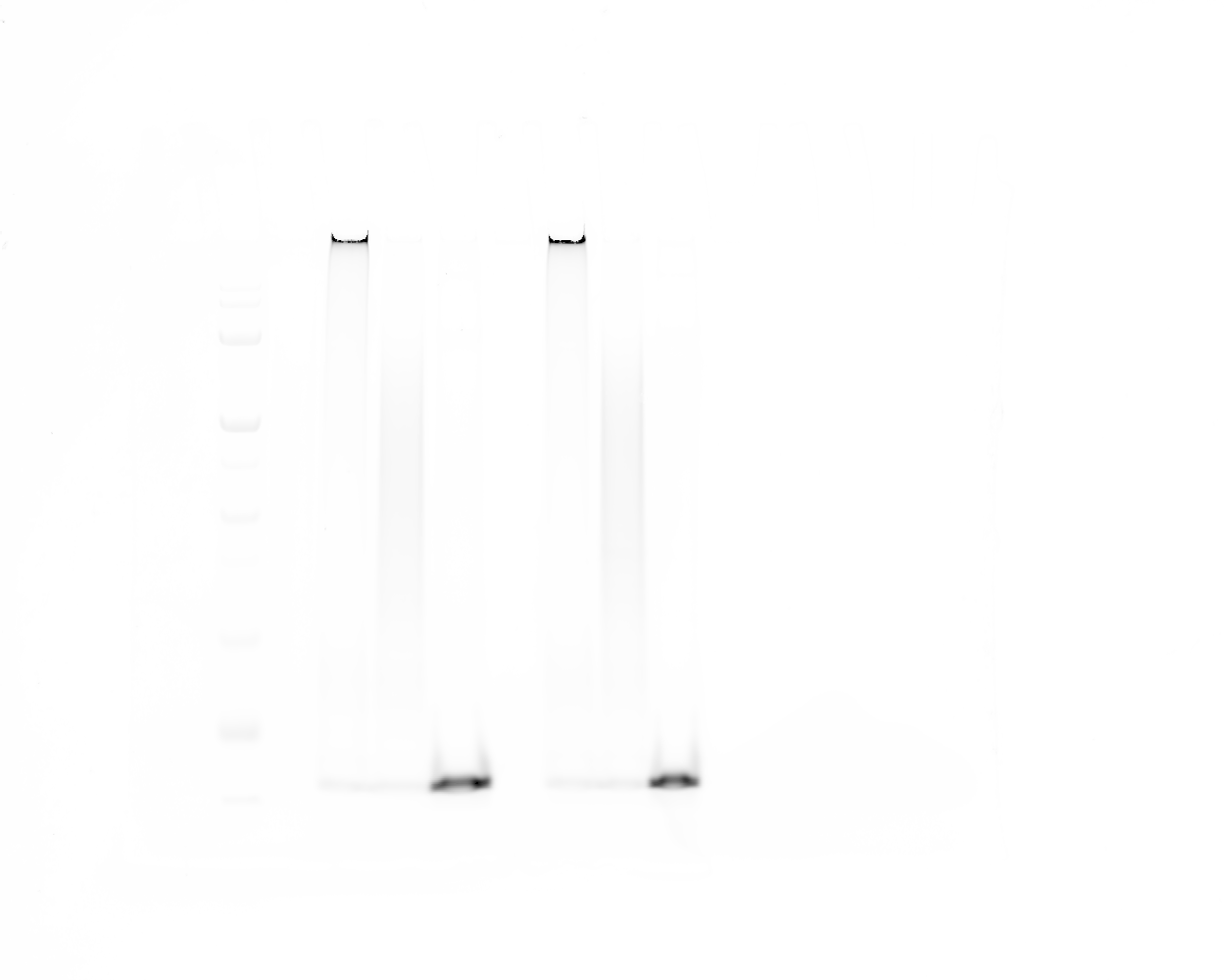

Supplement: Figure 1—source data 1. [file elife-61525-fig1-data1.zip › Figure 1 - panel B - Atto647 fluorescence.tif]

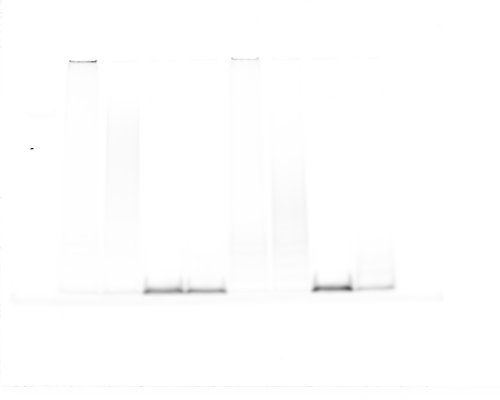

Supplement: Figure 1—figure supplement 3—source data 1. [file elife-61525-fig1-figsupp3-data1.zip › Figure 1 - figure supplement 3 - panel B - Atto550 fluorescence.tif]

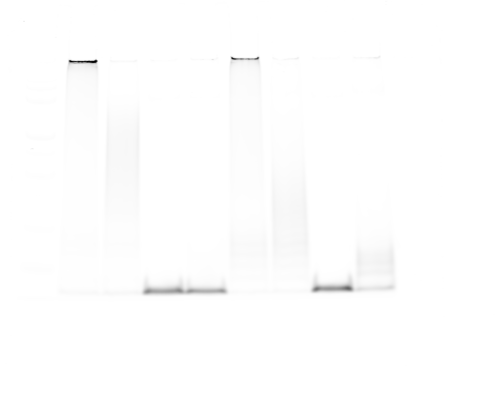

Supplement: Figure 1—figure supplement 3—source data 1. [file elife-61525-fig1-figsupp3-data1.zip › Figure 1 - figure supplement 3 - panel B - Atto647 fluorescence.tif]

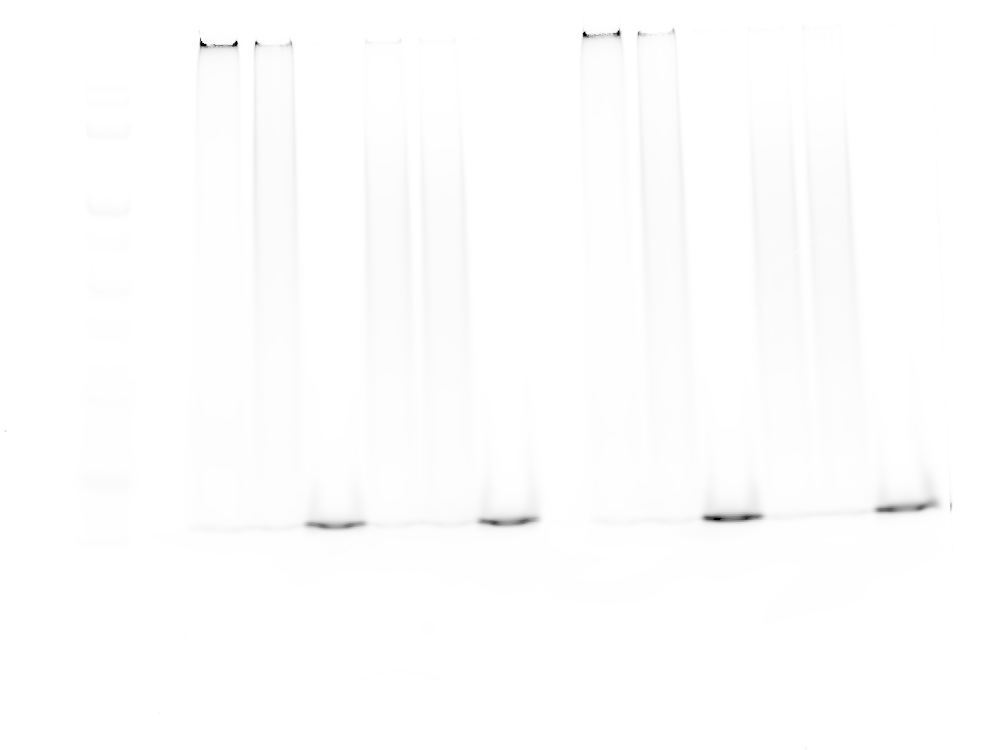

Supplement: Figure 2—source data 1. [file elife-61525-fig2-data1.zip › Figure 2 - panel A - Atto647 fluorescence.tif]

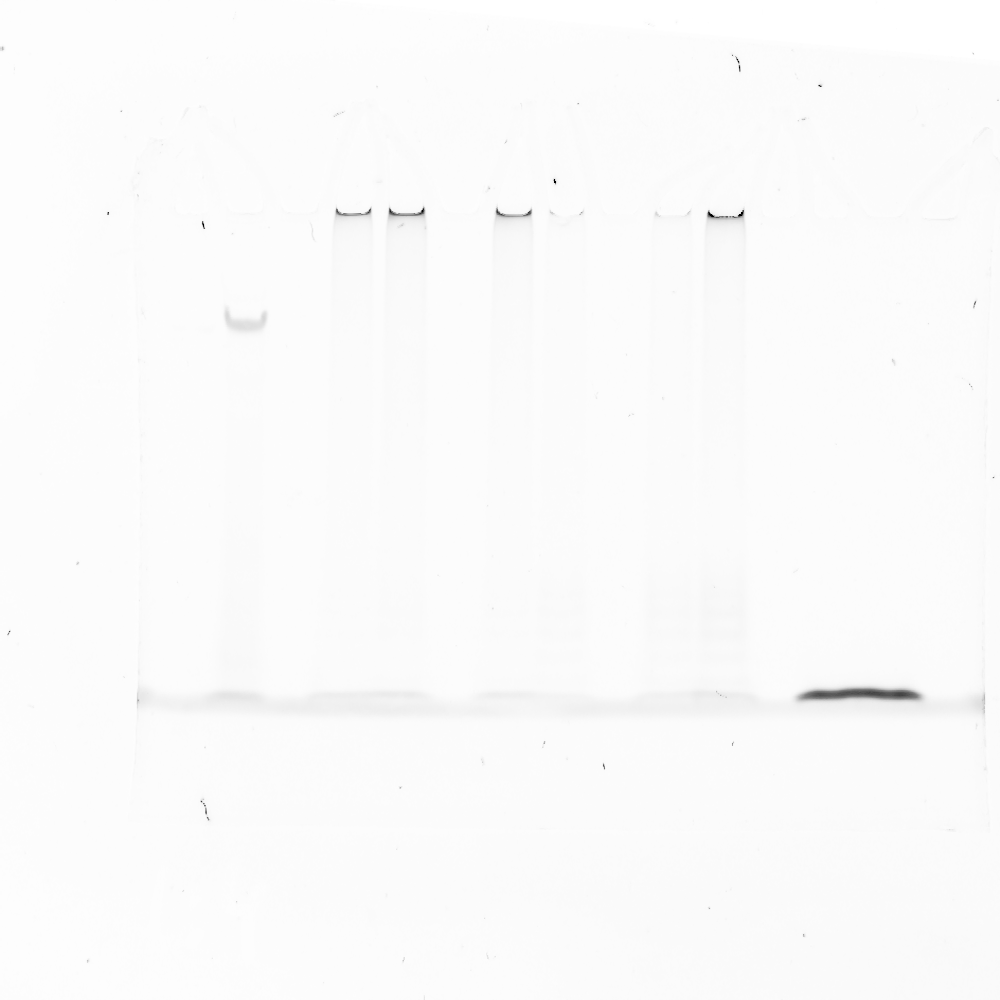

Supplement: Figure 2—figure supplement 1—source data 1. [file elife-61525-fig2-figsupp1-data1.zip › Figure 2 - figure supplement 1 - 20pc - Atto550 fluorescence.tif]

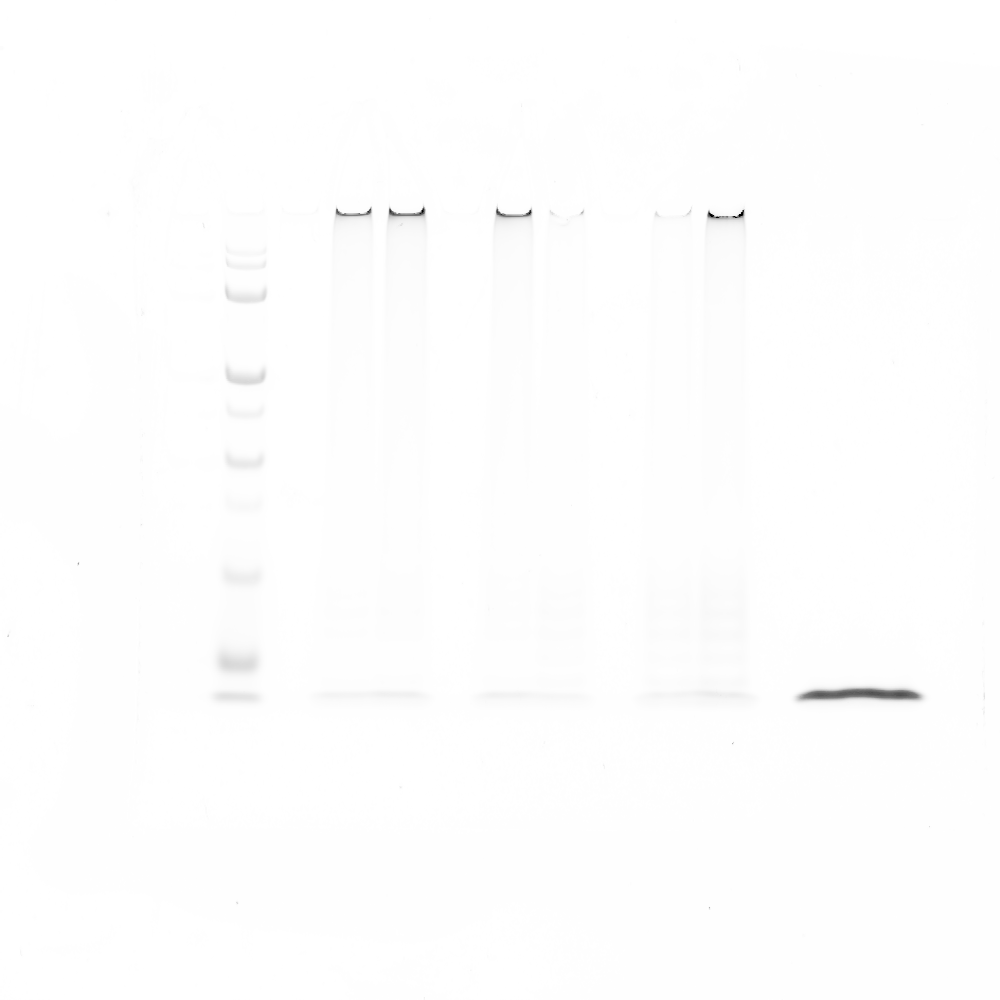

Supplement: Figure 2—figure supplement 1—source data 1. [file elife-61525-fig2-figsupp1-data1.zip › Figure 2 - figure supplement 1 - 20pc - Atto647 fluorescence.tif]

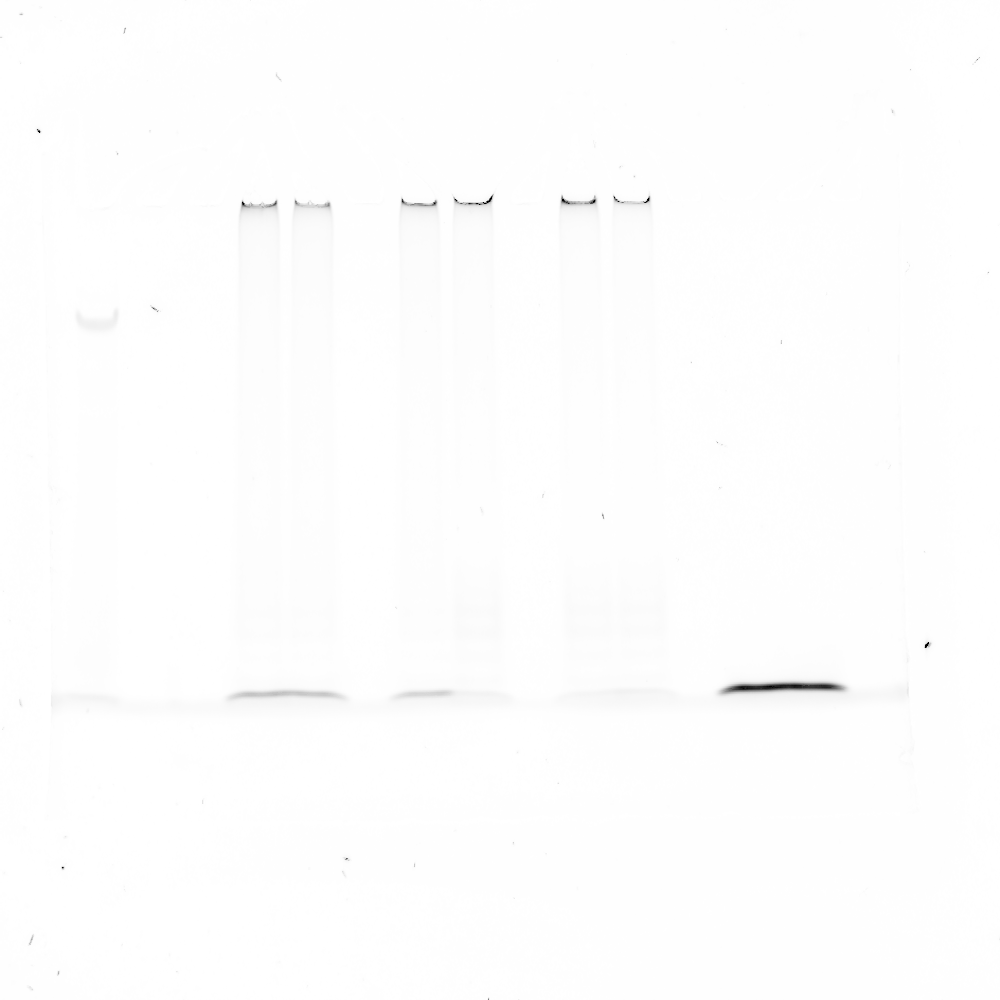

Supplement: Figure 2—figure supplement 1—source data 1. [file elife-61525-fig2-figsupp1-data1.zip › Figure 2 - figure supplement 1 - 50pc - Atto550 fluorescence.tif]

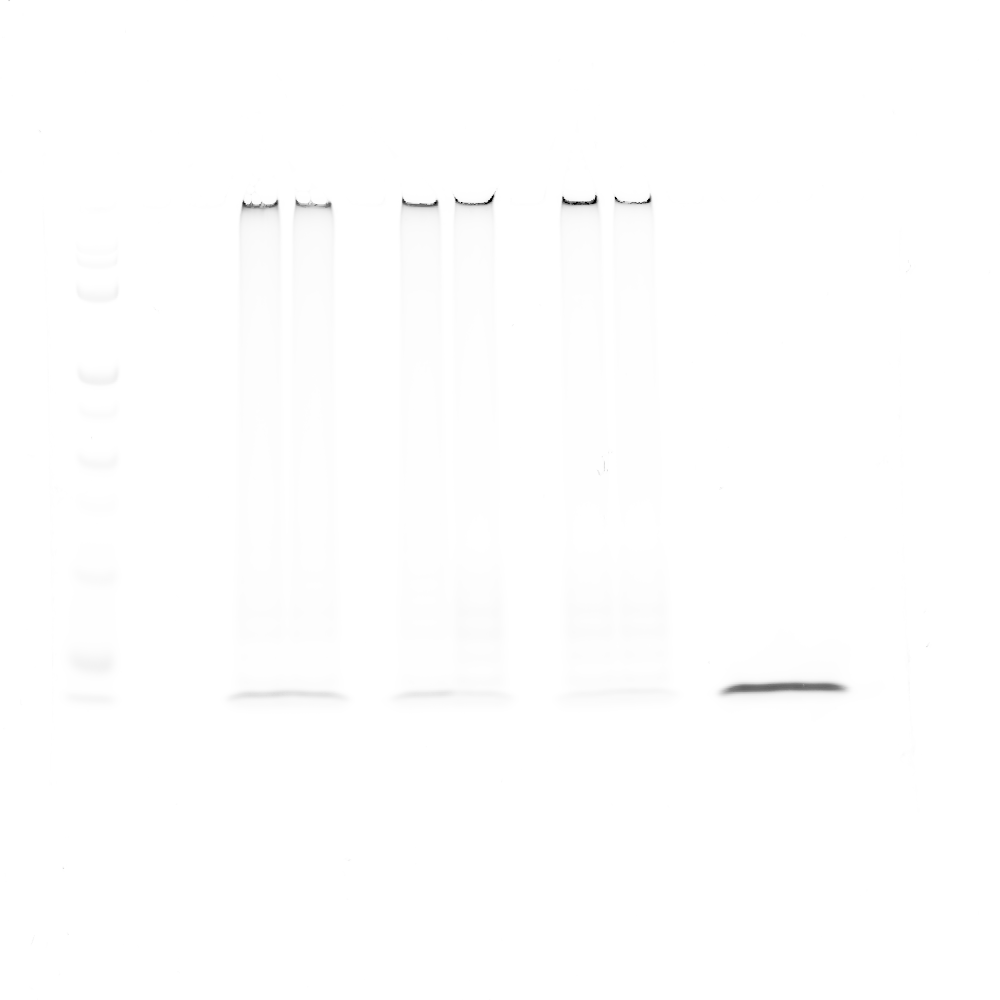

Supplement: Figure 2—figure supplement 1—source data 1. [file elife-61525-fig2-figsupp1-data1.zip › Figure 2 - figure supplement 1 - 50pc - Atto647 fluorescence.tif]

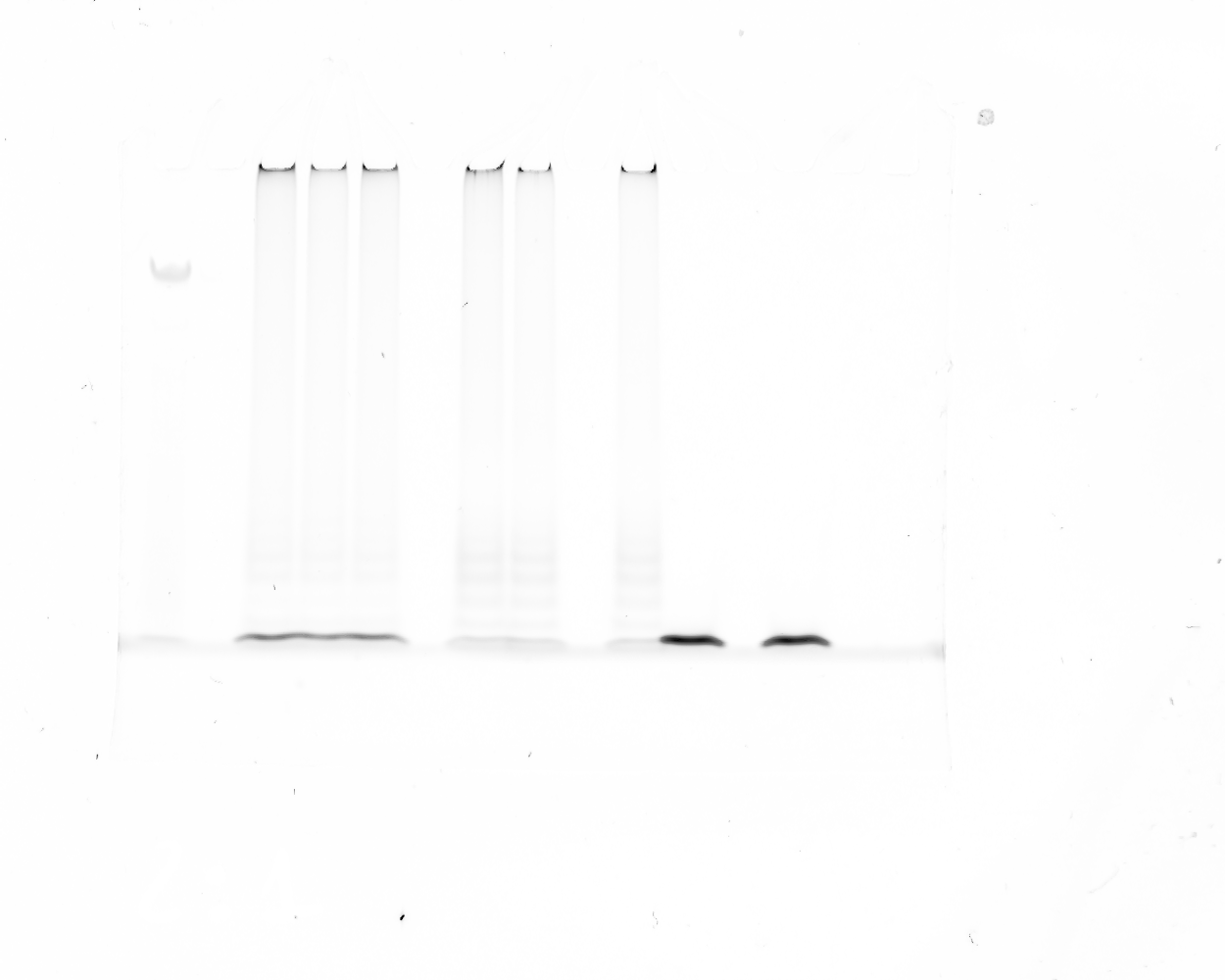

Supplement: Figure 2—figure supplement 1—source data 1. [file elife-61525-fig2-figsupp1-data1.zip › Figure 2 - figure supplement 1 - 67pc - Atto550 fluorescence.tif]

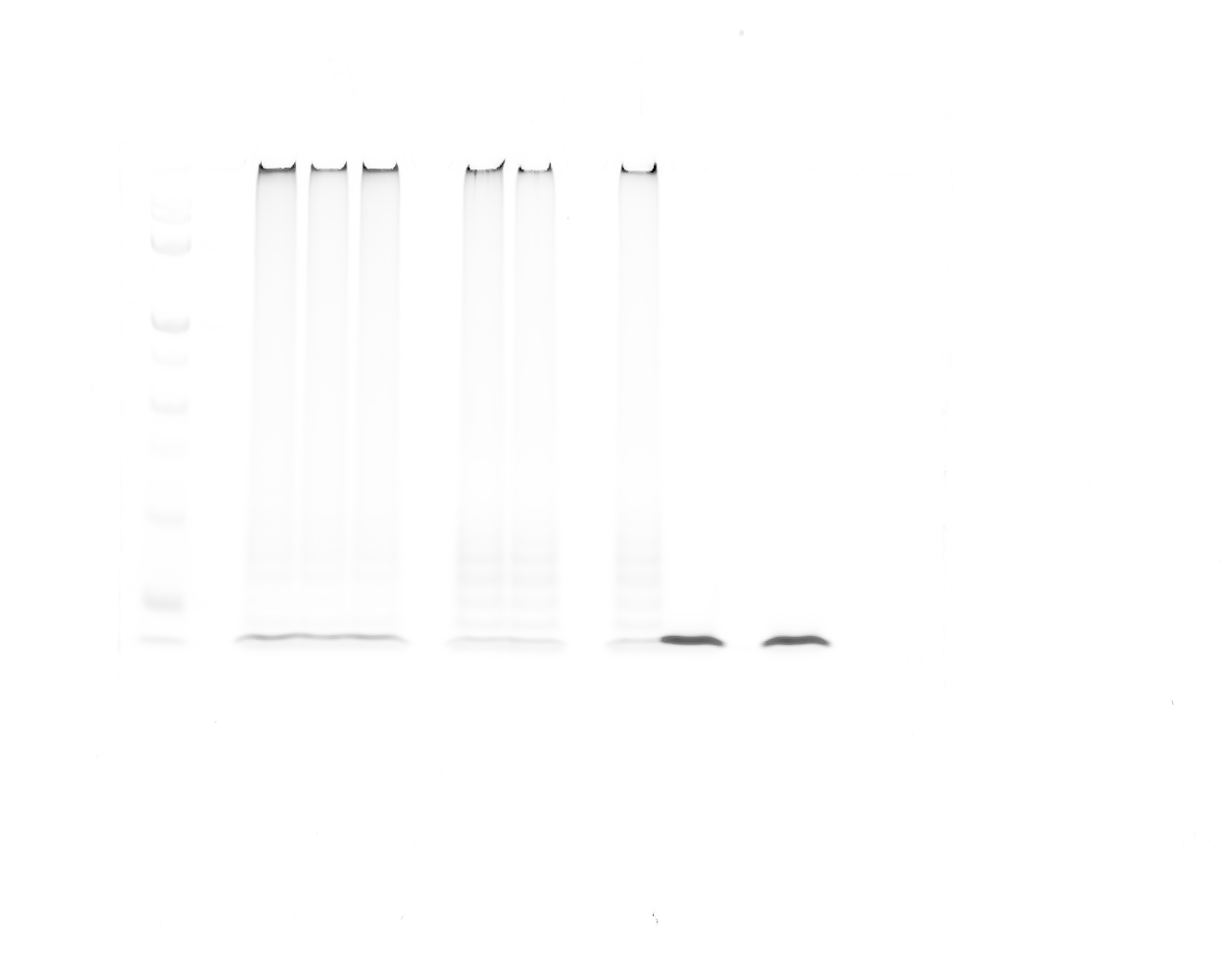

Supplement: Figure 2—figure supplement 1—source data 1. [file elife-61525-fig2-figsupp1-data1.zip › Figure 2 - figure supplement 1 - 67pc - Atto647 fluorescence.tif]

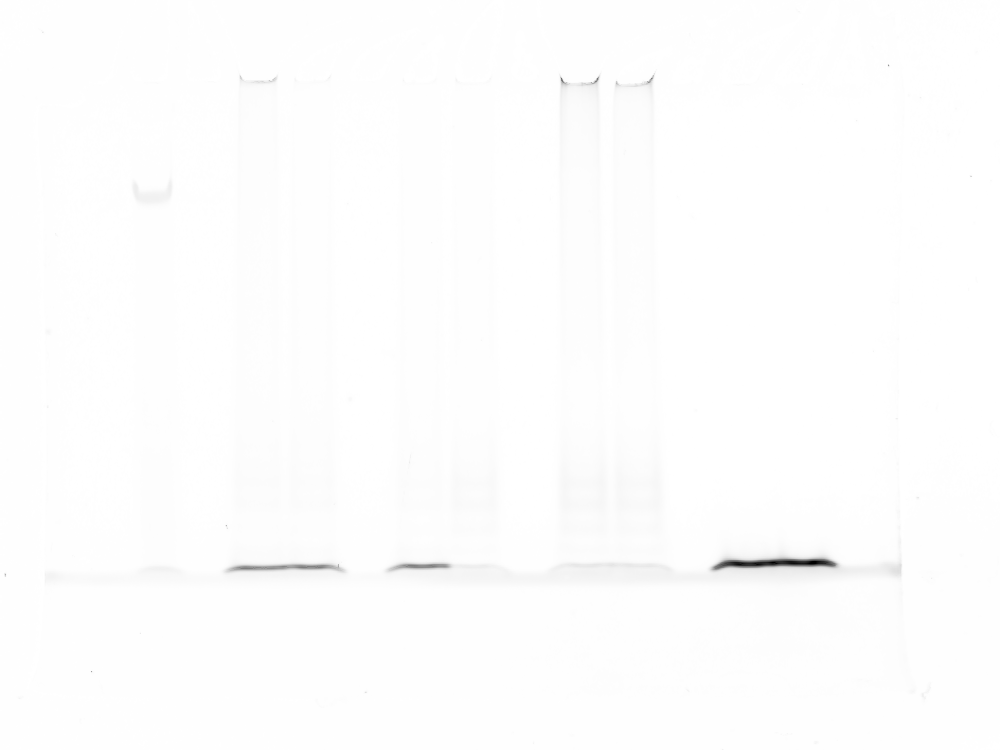

Supplement: Figure 2—figure supplement 1—source data 1. [file elife-61525-fig2-figsupp1-data1.zip › Figure 2 - figure supplement 1 - 80pc - Atto550 fluorescence.tif]

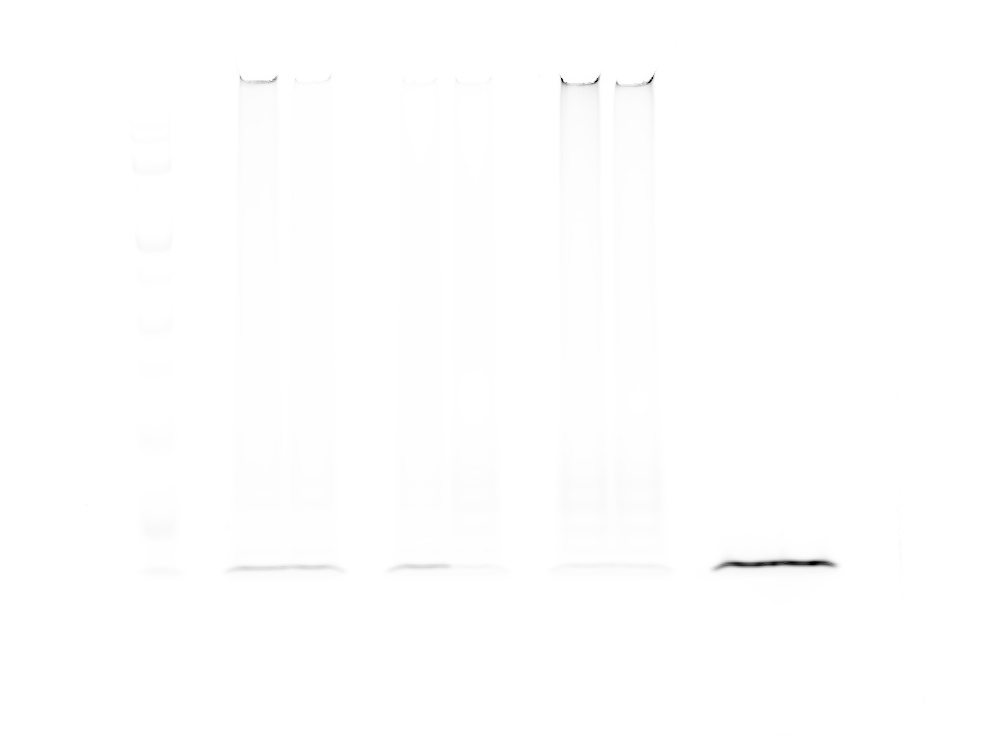

Supplement: Figure 2—figure supplement 1—source data 1. [file elife-61525-fig2-figsupp1-data1.zip › Figure 2 - figure supplement 1 - 80pc - Atto647 fluorescence.tif]

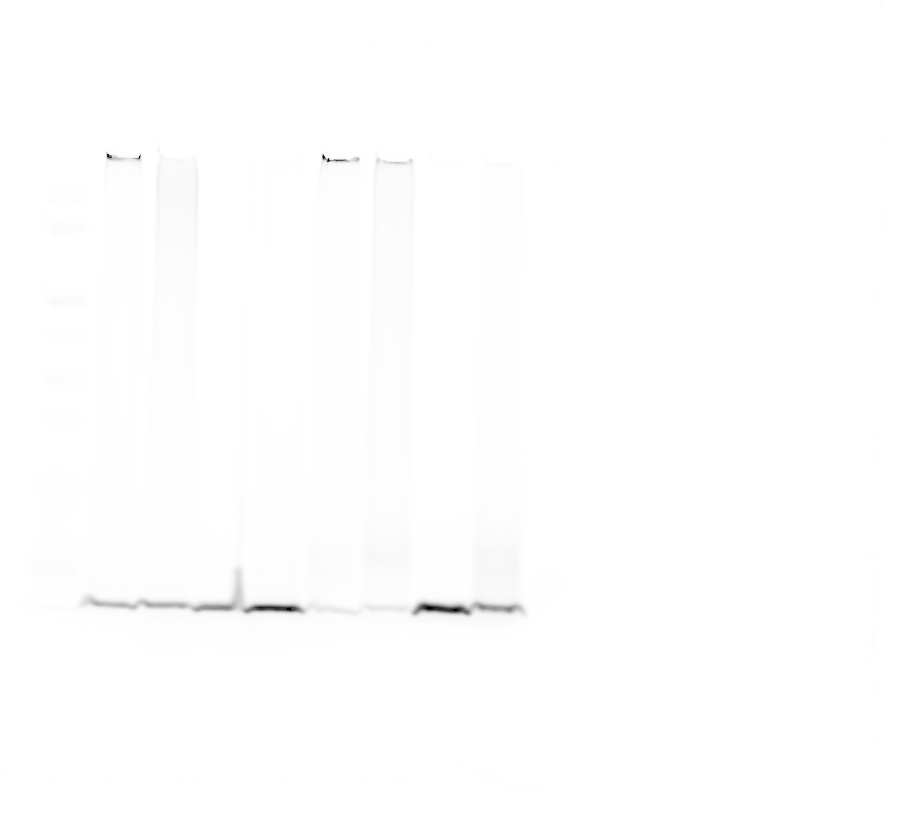

Supplement: Figure 3—source data 1. [file elife-61525-fig3-data1.zip › Figure 3 - panel C - Atto647 fluorescence.tif]

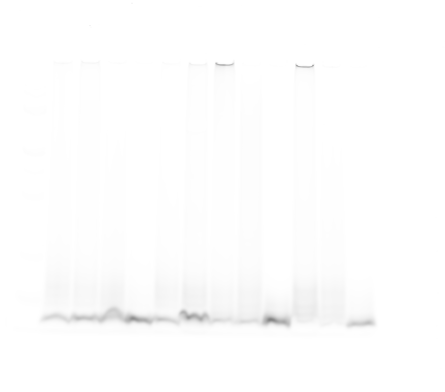

Supplement: Figure 3—source data 1. [file elife-61525-fig3-data1.zip › Figure 3 - panel E - Atto647 fluorescence.tif]

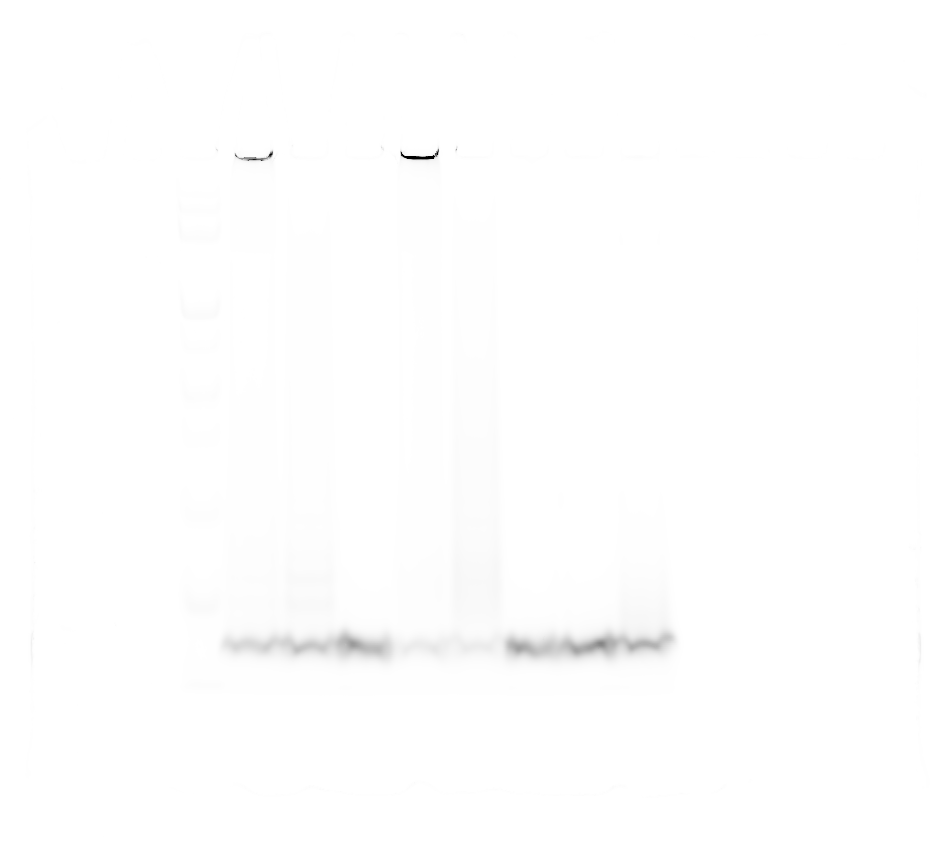

Supplement: Figure 3—source data 1. [file elife-61525-fig3-data1.zip › Figure 3 - panel G - Atto647 fluorescence.tif]

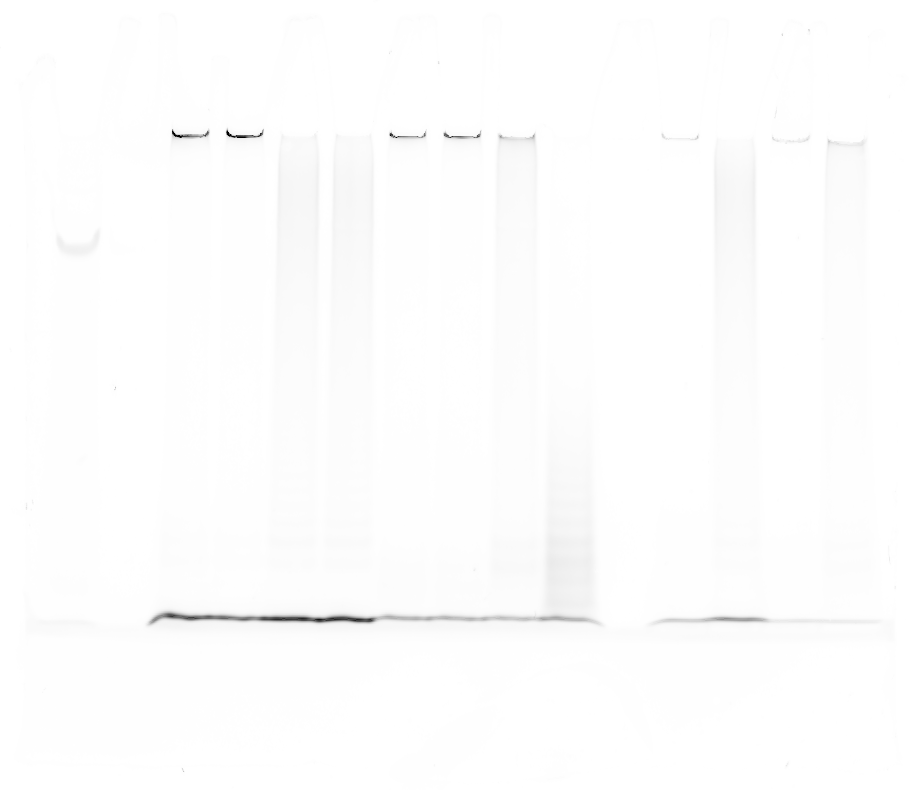

Supplement: Figure 3—figure supplement 2—source data 1. [file elife-61525-fig3-figsupp2-data1.zip › Figure 3 - figure supplement 2A - Atto550 fluorescence.tif]

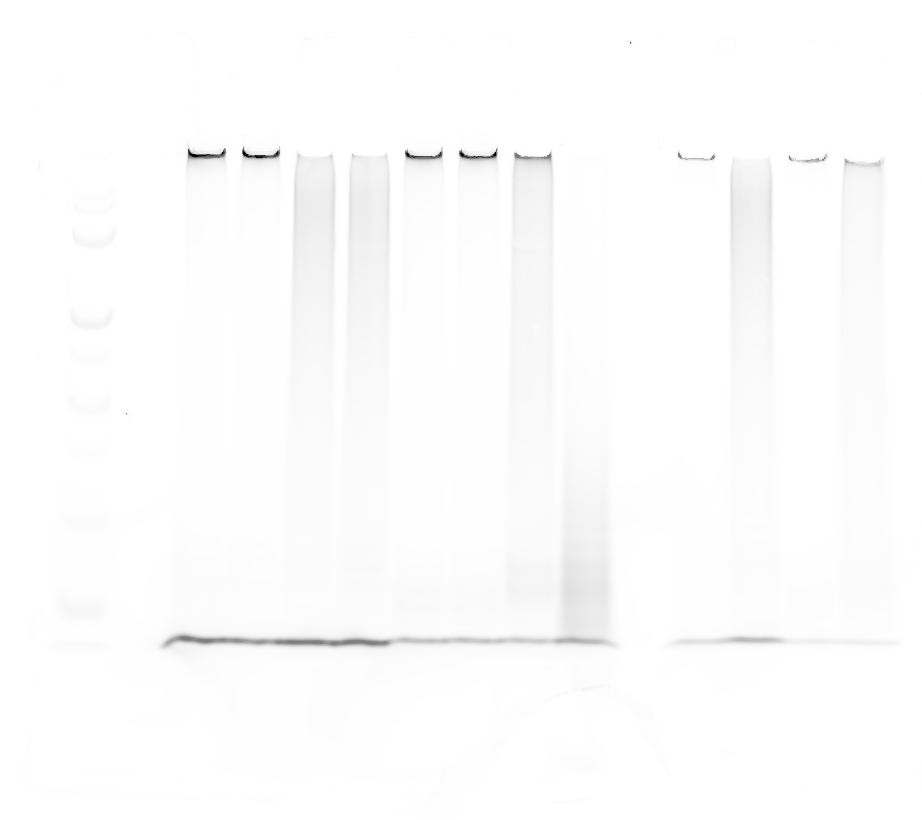

Supplement: Figure 3—figure supplement 2—source data 1. [file elife-61525-fig3-figsupp2-data1.zip › Figure 3 - figure supplement 2A - Atto647 fluorescence.tif]

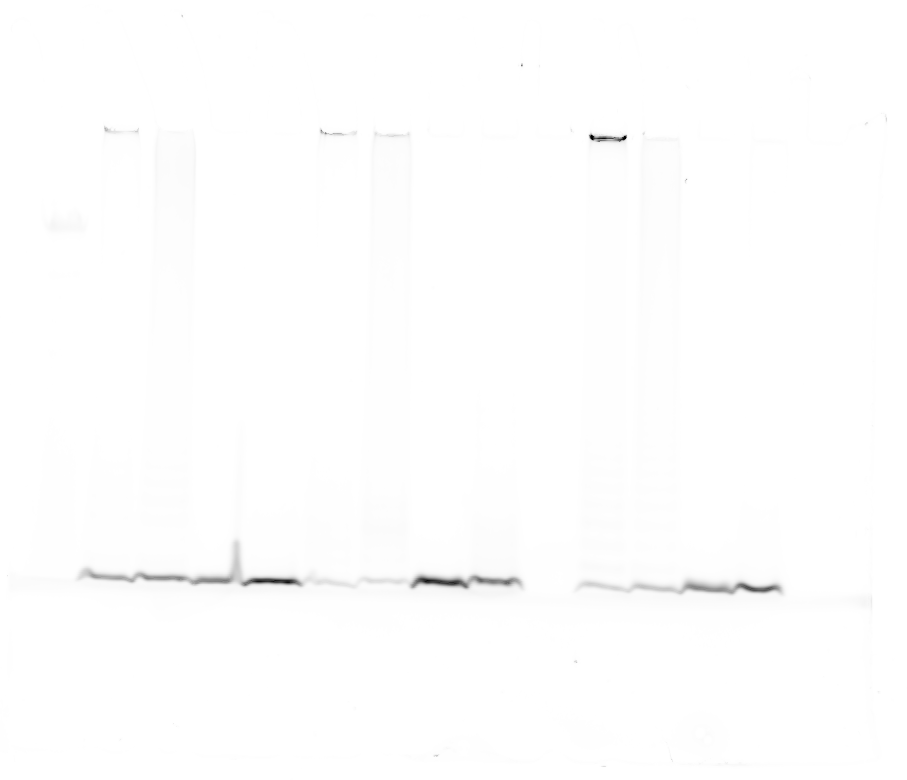

Supplement: Figure 3—figure supplement 2—source data 1. [file elife-61525-fig3-figsupp2-data1.zip › Figure 3 - figure supplement 2B - Atto550 fluorescence.tif]

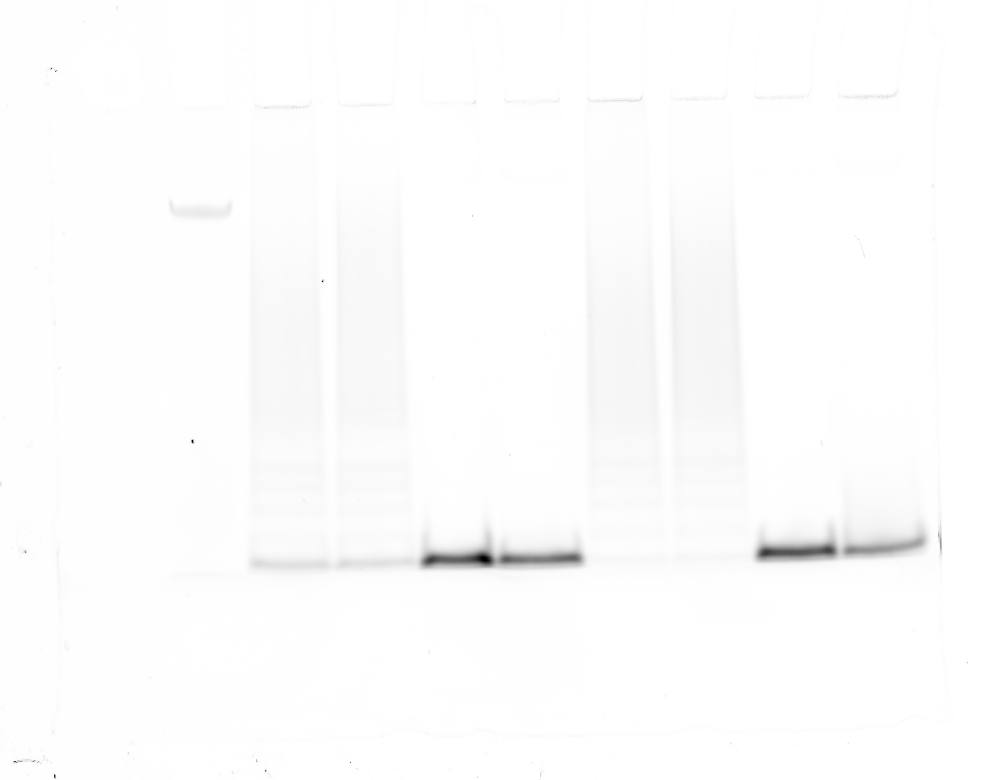

Supplement: Figure 3—figure supplement 7—source data 1. [file elife-61525-fig3-figsupp7-data1.zip › Figure 3 - figure supplement 7C - Atto550 fluorescence.tif]

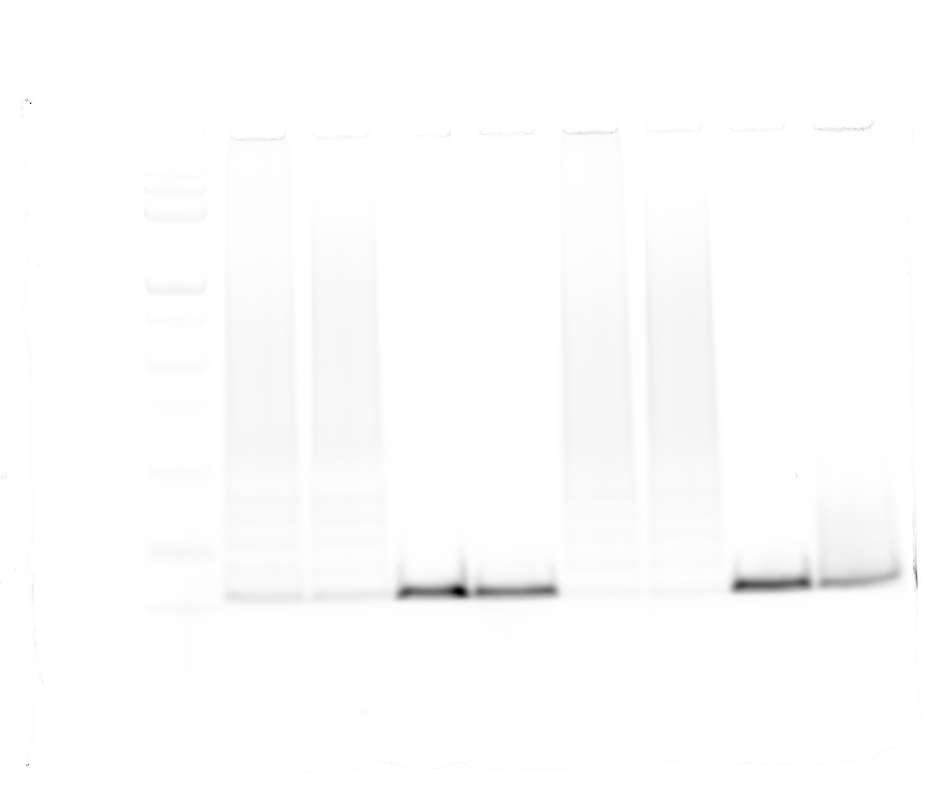

Supplement: Figure 3—figure supplement 7—source data 1. [file elife-61525-fig3-figsupp7-data1.zip › Figure 3 - figure supplement 7C - Atto647 fluorescence.tif]

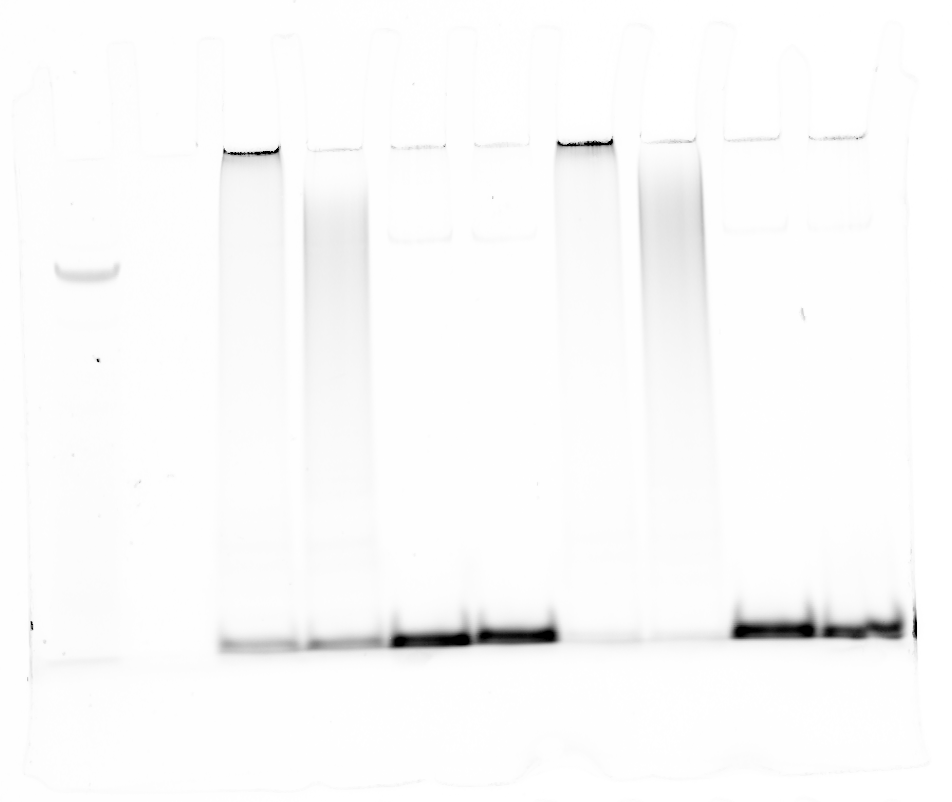

Supplement: Figure 3—figure supplement 8—source data 1. [file elife-61525-fig3-figsupp8-data1.zip › Figure 3 - figure supplement 8C - Atto550 fluorescence.tif]

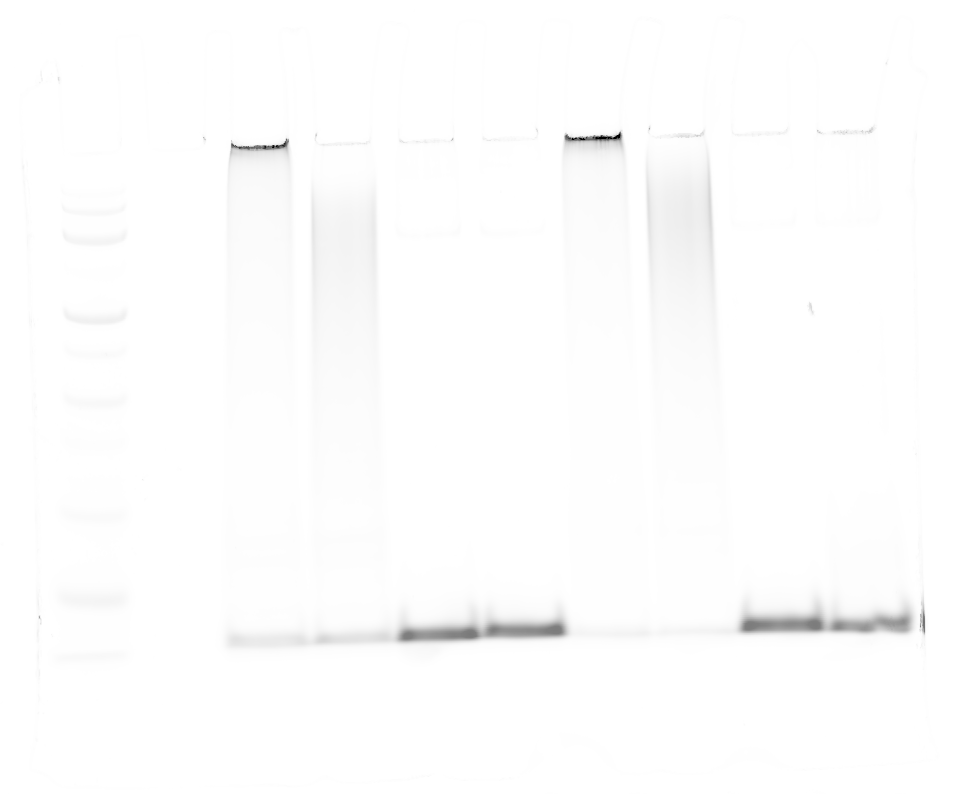

Supplement: Figure 3—figure supplement 8—source data 1. [file elife-61525-fig3-figsupp8-data1.zip › Figure 3 - figure supplement 8C - Atto647 fluorescence.tif]
