# Supplementary material for: Real-time monitoring of peptidoglycan synthesis by membrane-reconstituted penicillin-binding proteins
Source: eLife. 2021 Feb 24;10:e61525. doi: 10.7554/eLife.61525 (PMC7943195; doi:10.7554/eLife.61525)
Supplement: Supplementary file 1. [file elife-61525-supp1.docx]

**Supplementary Table 1. Oligonucleotides used in this work.**

| **Name** | **Sequence (5’ to 3’)** |
| --- | --- |
| PBP1B.Acineto-NdeI_f | AGATATCATATGATGAAGTTTGAACGTGGTATC GGTTTCTTC |
| PBP1B.Acineto-BamHI_r | GCGGGATCCTTAGTTGTTATAACTACCACTTGA AATG |
| Seq1_rev_PBP1B_Acineto | AGGTTCTAAACGGGCAACTC |
| Seq2_fwd_PBP1B_Acineto | TGGTTATGGATTGGCCTCTC |
| Seq3_fwd_PBP1B_Acineto | CTGGGCAAGCCAGATTGAAG |
| Seq4_fwd_PBP1B_Acineto | ACAATTACGCCAGAC ACCAG |
| PBP1B-MGC-F | CATCATCCATGGGCTGTGGCTGGCTATG GCTACTGCTA |
| PBP1B-CtermH-R | CATCATCTCGAGATTACTACCAAACATATCCTT |
| C777S-D | AACTTTGTTTCCAGCGGTGGC |
| C777S-C | GCCACCGCTGGAAACAAAGTT |
| C795S-D | CAATCGCTGTCCCAGCAGAGC |
| C795S-C | GCTCTGCTGGGACAGCGATTG |
